# Development of Peptide Mimics of the Human Acetylcholine Receptor Main Immunogenic Region for Treating Myasthenia Gravis

**DOI:** 10.3390/ijms26010229

**Published:** 2024-12-30

**Authors:** Vu B. Trinh, Robert H. Fairclough

**Affiliations:** 1 Department of Neurology, Davis School of Medicine, University of California, 1515 Newton Court, Davis, CA 95618, USA; vutrinh@ucdavis.edu; 2 Biochemistry, Molecular, Cellular, and Developmental Biology Graduate Group, University of California, Davis, CA 95618, USA; 3 Biophysics Graduate Group, University of California, Davis, CA 95618, USA

**Keywords:** immunotherapy, myasthenia gravis, main immunogenic region, human acetylcholine receptor

## Abstract

We have designed and produced 39 amino acid peptide mimics of the *Torpedo* and human acetylcholine receptors’ (AChRs) main immunogenic regions (MIRs). These conformationally sensitive regions consist of three non-contiguous segments of the AChR α-subunits and are the target of 50–70% of the anti-AChR autoantibodies (Abs) in human myasthenic serum and in the serum of rats with a model of that disease, experimental autoimmune myasthenia gravis (EAMG), induced by immunizing the rats with the *Torpedo* electric organ AChR. These MIR segments covalently joined together bind a significant fraction of the monoclonal antibodies (mAbs) raised in rats against electric organ AChR. Many of these mAbs cross react with the rat neuromuscular AChR MIR and induce myasthenic symptoms when injected into naïve rats. The human MIR mimic peptide (H39MIR) is evolutionarily related to that of the *Torpedo* electric organ MIR mimic peptide (*T*39MIR) with eight amino acid differences between the two MIR mimics. The mAbs raised to the electric organ AChR MIR cross react with the human and scores of other species’ neuromuscular AChRs. However, the mAbs do not cross react with the H39MIR mimic attached to the N-terminus of an intein–chitin-binding domain (H39MIR-IChBD) even though they do bind to the *T*39MIR-IChBD construct. To account for this difference in binding anti-MIR mAbs, each of the eight human amino acids was substituted individually into the *T*39MIR-IChBD, and four of them were found to weaken mAb recognition. Substituting the corresponding four *Torpedo* amino acids individually and in combination into the homologous positions in H39MIR-IChBD makes chimeric human MIR mimic peptides (*T*/H39MIR), some of which bind anti-MIR mAbs and anti-MIR Abs from rat EAMG and human MG sera. The best mAb binding chimeric peptide constructs may potentially serve as the basis of a diagnostic anti-MIR Ab titer assay that is both prognostic and predictive of disease severity. Furthermore, the best peptides may also serve as the targeting element of a non-steroidal antigen-specific treatment of MG to remove anti-AChR MIR Abs, either as fused to the N-terminals of the human immunoglobin F_c_ fragment or as the targeting component of a T cell chimeric autoantibody receptor (CAAR) directed to anti-MIR memory B cells for elimination.

## 1. Introduction

Myasthenia gravis (MG) is an antibody-mediated autoimmune disease for which a strong case can be made for developing a rational non-steroidal antigen-specific therapy as a potential pathway to a cure. The autoantibody-mediated attack is directed to the neuromuscular junction’s (NMJ’s) acetylcholine receptors (AChRs), resulting in the simplification of the folded post-synaptic membrane with a concomitant decrease in AChR density in that membrane [1,2]. The result is the debilitating use-induced fatigue of the affected patient muscles.

Many monoclonal antibodies (mAbs) derived from rats immunized with electric organ AChRs bind to the *Torpedo* electric organ AChR (*T*AChR) and mutually block one another’s binding [3,4,5,6]. Likewise, Whiting et al. have produced mAbs in mice from five partially overlapping regions of the human AChR (HAChR) [7]. When the mutually blocking electric organ mAbs are purified and injected into naïve rats, they cross-react with the rat neuromuscular AChR and cause MG symptoms [8,9,10]. In addition, many of these mAbs bind to the neuromuscular AChRs of numerous species, including those of humans [6,11], where they block the binding of 50–70% of the anti-AChR antibodies (Abs) in MG sera [11]. These mutually blocking mAbs bind to what appears to be an immunological hot spot called the main immunogenic region (MIR) [12,13]. Electron micrographs of frozen hydrated 2D crystalline arrays of *T*AChRs, decorated with single chain Fv or Fab fragments of anti-MIR mAb 35, detected the MIR at the crest of the extracellular region of each of the two AChR α subunits [14].

**Figure 1:**
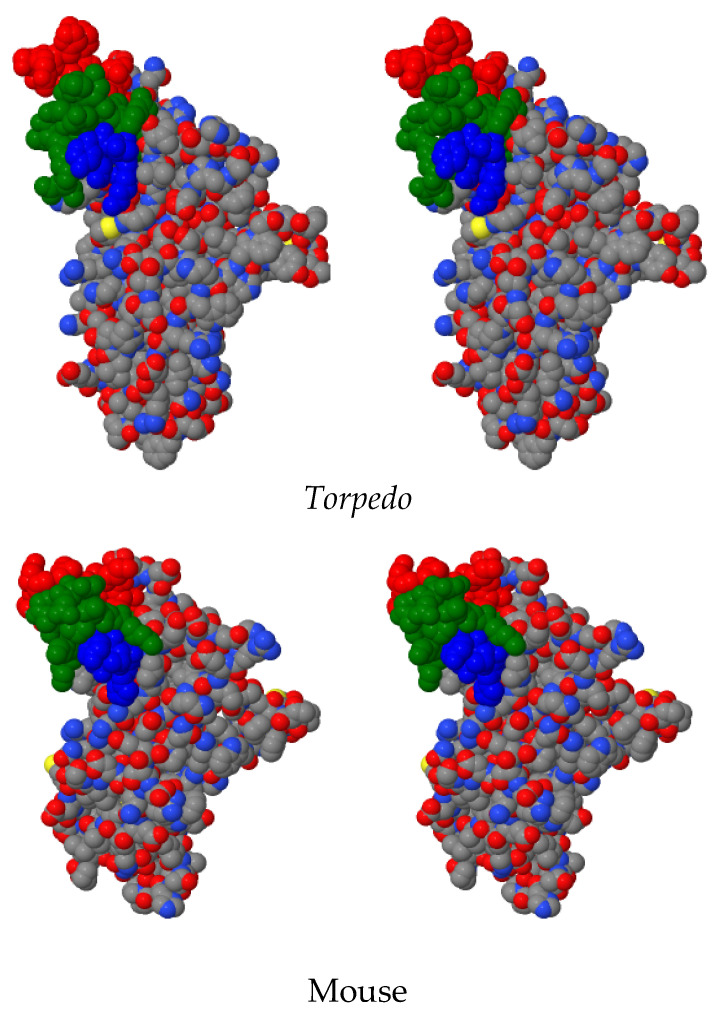
Stereo view (wall-eyed viewing) of the *Torpedo* and Mouse α subunit ECD (α1–211). These extracellular domains are cut from the 4.0 Å refined EM structure of the *Torpedo* AChR [16] and the 1.94 Å refined X-ray crystal structure of the mouse AChR α1 ECD [18] with CPK color coding. The three MIR segments located at the tops of the ECD are color coded red-α(1–12), green-α(65–79), and blue-α(110–115). For orientation, the cys 192–193 disulfide associated with the acetylcholine binding site is positioned at the farthest loop to the right of the models.

To treat MG, unlike any of the existing therapies, we hope to develop an MG-specific therapy that eliminates only the pathogenic anti-AChR MIR directed Abs active in MG and the B cells that possess the genetic plans for producing these Abs. Current immune-directed therapies of MG affect the entire immune system, which results in the potential loss of critical protection against bacterial and viral infections and weakens existing immunizations. Hence, our anti-AChR MIR construct may provide this very specific therapy without compromising an individuals’ immune system protection against any pathogenic invasion. To target the MIR, we used anti-MIR mAb 132A, raised in rats against *T*AChR, to further map the MIR epitope to three non-contiguous segments of the *T*AChR α subunit: α(1–12), α(65–79), and α(110–115) [15], providing significant insights into the composition and structural organization of the MIR. Locating these segments on Unwin’s 4Å refined 3D model of the *T*AChR (PDB 2BG9) reveals their close proximity to one another as adapted in Figure 1 from Unwin’s 4Å refinement [16]. From these data, we designed, synthesized, and characterized a 39 amino acid (aa) peptide mimic of the *Torpedo* MIR (*T*39MIR) consisting of the three non-contiguous segments of the AChR α subunit joined by flexible gly–ser (G-S) linkers, and then fused to the amino terminus of an intein–chitin-binding domain (*T*39MIR-IChBD) [17]. mAb 132A binds to the *T*39MIR-IChBD construct with high affinity with a K_d_ of 2.1 × 10^−10^ M compared to a K_d_ of 3.4 × 10^−10^ M for mAb 132A complexed to the native *Torpedo* AChR. This peptide construct is also the target of anti-MIR mAbs 35 and 334 raised against electric organ AChR, and mAb 198 raised against the human neuromuscular AChR. The *T*39MIR-IChBD is also the target of MIR-directed Abs from the serum of rats with experimental autoimmune myasthenia gravis (EAMG), the animal model of MG induced by the immunization of the rats with purified *Torpedo* AChR [17]. Removing these EAMG anti-MIR Abs lays the groundwork for a potential therapeutic treatment of human MG. However, subsequent experiments revealed that few, if any, Abs from human MG sera bind to *T*39MIR-IChBD, despite anti-AChR MIR mAbs raised to the electric organ AChR binding to the MIR regions of intact human neuromuscular AChR. Hence the need for an MIR mimic peptide to bind to anti-AChR MIR Abs in the sera of MG patients.

**Figure 2:**
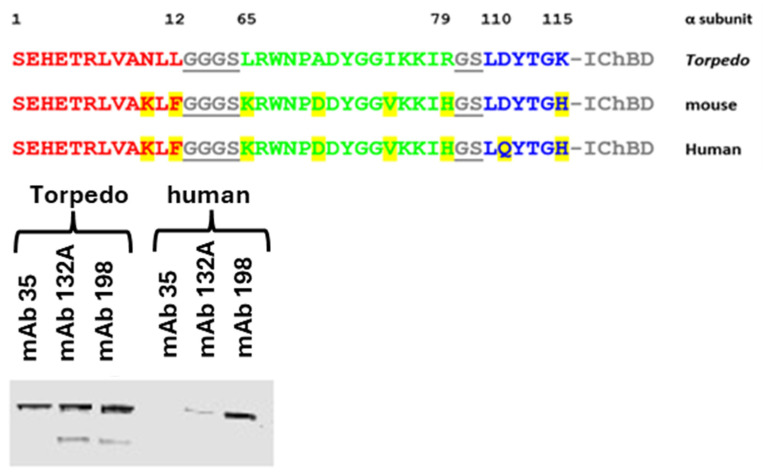
(**Upper**) *Torpedo*, mouse, and human 39MIR peptides joined to an IChBD. Color code: grey and underlined GS linkers, red-α(1–12), green-α(65–79), blue-α(110–115). Yellow highlights residue positions that differ between *Torpedo* and rat/mouse and human sequences. (**Lower**) Pull-out of mAbs 35, 132A, and 198 by *Torpedo* and Human 39MIR mimics. The mimics are fused to IChBDs and adsorbed to chitin beads. Mabs are added and the mAb-peptide-IChBD-Chitin Beads complexes are pelleted and taken up into an SDS sample buffer and run on a 7.5% SDS gel. The gel-separated proteins are then transferred to nitrocellulose and the mAbs are stained with IRDye 680 goat anti-rat IgG. mAb 35 does not bind to the human peptide mimic, whereas 132A binds weakly to the human mimic compared to its binding to the *Torpedo* mimic, and 198 seems to bind reasonably well to both *Torpedo* and human 39MIR mimics.

We have now produced the equivalent human MIR mimic, H39MIR-IChBD, starting with the sequence of the homologous human three MIR segments corresponding to those of the *T*39MIR-IChBD segments (Figure 2). In this human peptide sequence, there are eight amino acid (aa) differences from the corresponding 39 aa *Torpedo* sequence. We demonstrate that this human construct is not recognized by the anti-MIR mAbs that bind to the *T*39MIR-IChBD construct, nor is it recognized by anti-MIR directed Abs in MG serum. The current work builds on mutational analysis of the H39MIR-IChBD to develop a peptide that does bind to the anti-MIR mAbs, and potentially to the human MG sera anti-MIR Abs. A refined best possible MIR mimic peptide can then be used to assay for anti-MIR Ab titer, as well as to construct a biologic to treat rat EAMG and human MG.

## 2. Results

### 2.1. Binding of Anti-MIR mAbs to MIR Mimic Peptides T39MIR-IChBD and H39MIR-IChBD

The *Torpedo* and human AChR MIR peptide mimics (*T*39MIR and H39MIR, respectively) were made by linking the segments α(1–12), α(65–79), and α(110–115) of their α-subunits with gly–ser (G-S) linkers and covalently coupling the peptides to IChBDs (Figure 2). The segments in the respective *Torpedo* and the mouse mammalian AChR α subunits’ extracellular domain are depicted and compared in stereo in Figure 1, with the three respective segments colored red, green, and blue in the two space filling displays. The *Torpedo* and the human peptides differ from each other by eight aas (Figure 2). Both peptide mimics were tested for binding mAbs 35 and 132A, two anti-electric organ AChR MIR mAbs, and mAb 198, an anti-human neuromuscular AChR MIR-directed mAb. With this test, an attempt was made to extract each mAb using the peptide mimics fused to an IChBD adsorbed to chitin beads (Figure 2). Comparing the extraction of mAbs 35, 132A, and 198 by the *T*39MIR and H39MIR-IChBD constructs suggests that the mAbs greatly prefer binding to the *Torpedo* mimic, even though mAb 198 derives from the immunization of a rat with human AChR (Figure 2). In each case, more mAb is pulled out by the *T*39MIR construct than by the H39MIR construct. Additionally, mAb 35 is not pulled out at all by the H39MIR construct in this assay, in which the folded 39MIR constructs have not been denatured until after the mAbs have had a chance to bind to the mimic peptide constructs and the complexes pelleted via the attached chitin beads and loaded onto the SDS gel.

### 2.2. Western Blots to H39MIR: mAb 198 Binds Weakly, but Neither mAb 35 nor 132A Bind

**Figure 3:**
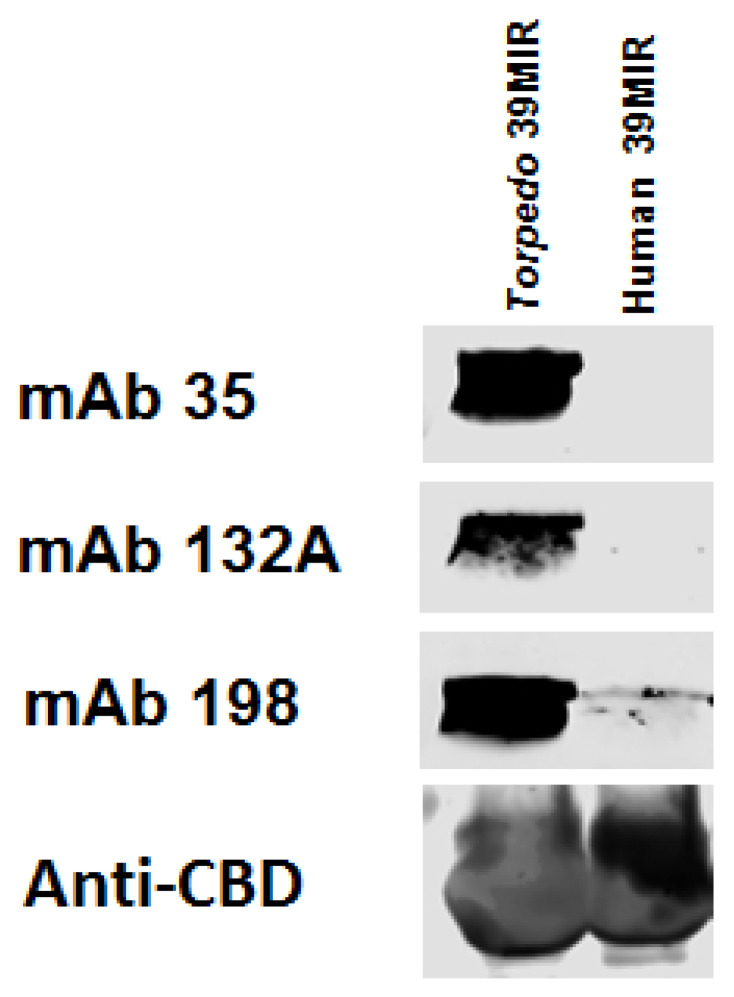
Western blots of the *T*39MIR-IChBD and H39MIR-IChBD mimics. Chitin beads adsorbed with the mimics, were collected and run on an SDS-PAGE gel, transferred to a nitrocellulose membrane, and analyzed for binding mAbs 35, 132A, 198 and an anti-ChBD mAb was detected with IRDye 680 goat anti-rat IgG.

mAb 198 recognizes both *T*39MIR-IChBD and H39MIR-IChBD in the pull-out assay, but in Western blots (Figure 3), mAb 198 binds ~10 times less mAb to the H39MIR construct than to the *T*39MIR construct (top two lines in Table 1). A small fraction of the H39MIR construct presumably refolds after SDS solubilization to a state similar enough to the native MIR to be only very weakly recognized by mAb 198, but not similar enough for binding mAbs 35 nor 132A (Figure 3) whereas all three mAbs bind well to the *T*39MIR construct. This *Torpedo* construct refolds better after SDS solubilization than the H39MIR construct. A large fraction of the H39MIR construct seems to not refold to a native structure. These results suggest that the eight aa differences between *Torpedo* and human MIR mimic peptides not only change the charge on three of the aas, but also cause a large fraction of the H39MIR mimics to refold after SDS denaturation to a slightly different MIR structure from that of the *T*39MIR mimic and that of the native human neuromuscular AChR MIR.

### 2.3. Effects of the Individual Human Aa Mutations on the Torpedo 39MIR Mimic

**Figure 4:**
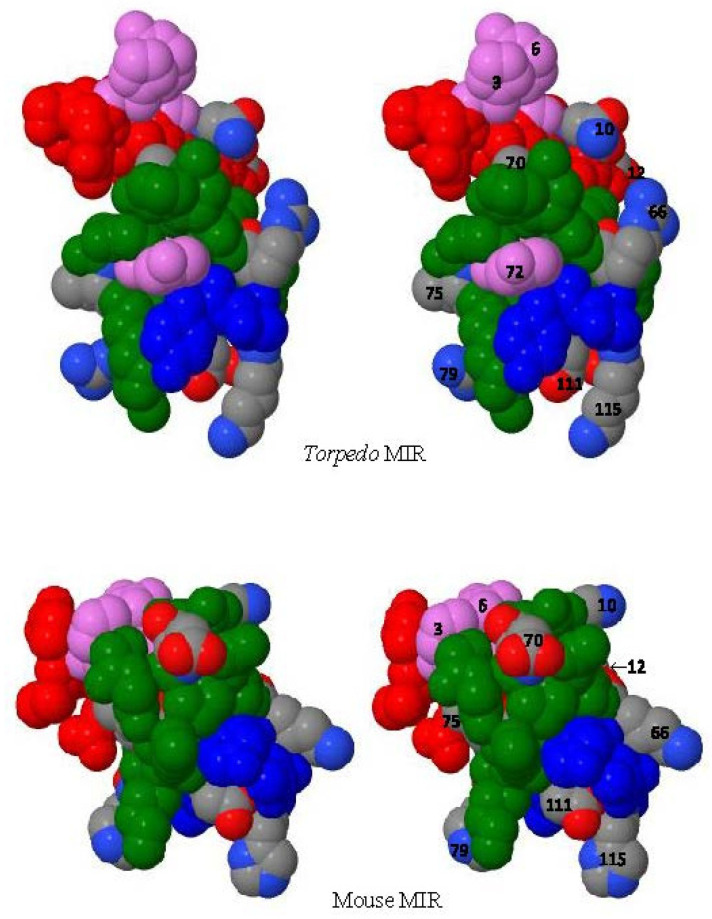
Stereo view (wall-eyed viewing) of the structures of the *Torpedo* and mammalian MIRs. The *Torpedo* structure is from Unwin, 2005 [16], and the mouse structure is from Dellisanti et al., 2007 [18]. The seven aas that differ between the two structures area displayed in CPK color format (as well as D111 which is the same in both structures) on a background of α(1–12) red, α(65–79) green, α(110–115) blue, and α(3,6,72) violet. Note the change in packing of aas 3, 6, and 72 in the two structures. Y72 in the mammalian structure is totally buried behind D70, whereas in the *Torpedo* structure it is prominently exterior.

**Figure 5:**
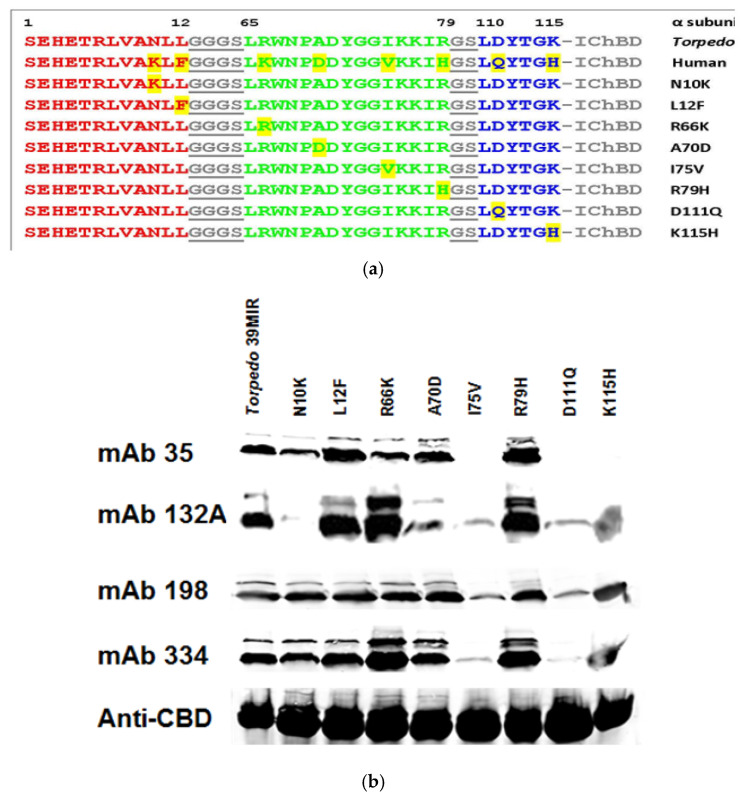
(**a**) Sequences of the *T*39MIR and H39MIR mimics and the *T*orpedo mutants joined to IChBDs. Color code: grey and underlined GS linkers, red-α(1–12), green-α(65–79), blue-α(110–115). Yellow highlights human residues that differ from those in the *Torpedo* sequence. (**b**) Western blots of the single-point mutated peptides of the *T*39MIR. Chitin beads adsorbed with the *T*39MIR and various mutant *T*39MIR peptide-IChBD fusion proteins expressed in *E. coli* transfected with the constructs encoded in pTYB1were all run on an SDS-PAGE gel, transferred to a nitrocellulose membrane, and analyzed for binding mAbs 35, 132A, 198, 334 and an anti-ChBD mAb detected with IRDye 680 anti-rat IgG. We suspect that the second narrow band above the lower broader bands derives from the transfer of a peptide (4.2kDa) from the IChBD (via an intein-formed thioester) to a lysine ε amino on another MIR peptide-IChBD, which is potentially the peptide lysine at 115.

To assess the effect of each of the eight individual aa differences between the *Torpedo* and the human MIR mimics and the contribution of each aa to mAb recognition, we cloned eight *T*39MIR mutant peptides, each with one of the eight aas substituted from human H39MIR into the corresponding *Torped*o sequence. The location of those single aa substitutions into the *Torpedo* MIR mimic peptide, N10K, L12F, R66K, A70D, I75V, R79H, D111Q, and K115H, are illustrated in the Figure 4 stereo structural models as the numbered aas along with Y72. The sequences of the peptides themselves are presented in Figure 5a. These peptides, each attached to the N-terminal of an IChBD, were collected on chitin beads, solubilized in SDS, run on an SDS gel, transferred to nitrocellulose membranes, and analyzed via Western blots for anti-MIR mAb binding (Figure 5b) compared to the mAb binding to the *Torpedo* MIR mimic construct. mAbs 35, 132A, 198, and 334 all bind to *T*AChR and are MIR-directed mAbs. Anti-ChBD mAb detects the amount of each of the expressed peptide-IChBD constructs for comparison purposes.

In Western blots of the mutant *T*39MIR constructs compared to the unmodified *T*39MIR construct (Figure 5b and Table 1), mAb 35 does not bind to I75V, D111Q, nor to K115H mutants, and the N10K mutant binds only 29% of the amount of mAb 35 that binds to *T*39MIR; mAb 132A binds 20% and 40% to A70D and K115H mutants, respectively, of that which it binds to *T*39MIR, and 132A does not bind to N10K, and virtually does not bind to neither I75V nor D111Q; mAb 198 binds 16% to I75V and D111Q; and, finally, mAb 334C binds 59% and 42% to N10K and K115H, respectively, and virtually does not bind to I75V and D111Q. mAb 147G, the negative control raised against fluorescein, did not bind to any of the peptides. All four anti-MIR mAbs bind poorly, if at all, to the I75V and D111Q mutants compared to their binding to *T*39MIR. In addition, the quantitative ratios of stain densities displayed in Table 1 show that some of the mutations actually enhance the binding of several of the mAbs compared to their binding to the unmutated peptide.

**Table 1:** Quantitative summary of Western blots of site-directed human aa mutants of the *T*39MIR-construct. (- no binding). Each entry for a particular mutant *T*39MIR-construct is the ratio of the mAb stain density to that of the unmutated *T*39MIR-construct in Figure 5b. Note that some mutants stain better than the unmutated construct.

Mutant	mAb 35	mAb 132A	mAb 198	mAb 334C
*T*39MIR	1.00	1.00	1.00	1.00
N10K	0.29	0.03	0.82	0.59
L12F	1.42	1.10	0.90	0.88
R66K	0.62	1.55	1.03	2.75
A70D	0.93	0.20	1.08	1.04
I75V	-	0.06	0.16	0.03
R79H	1.91	1.65	0.97	2.40
D111Q	-	0.07	0.16	0.02
K115H	-	0.40	1.07	0.42

In summary, the binding of all four of the anti-MIR mAbs are dramatically impaired by the I75V and D111Q mutations, and the N10K and A70D mutations most noticeably affect mAb 132A binding. As a result of the study of the eight aa differences between the *T*39MIR and the H39MIR, the four human aas at 10, 70, 75, and 111 were identified as the most detrimental to anti-MIR mAb binding to H/T39MIR chimeric mimics, and coincidentally include the three aas differing in charge between the two sequences.

### 2.4. Optimization of a Torpedo/Human Chimeric 39MIR

**Figure 6:**
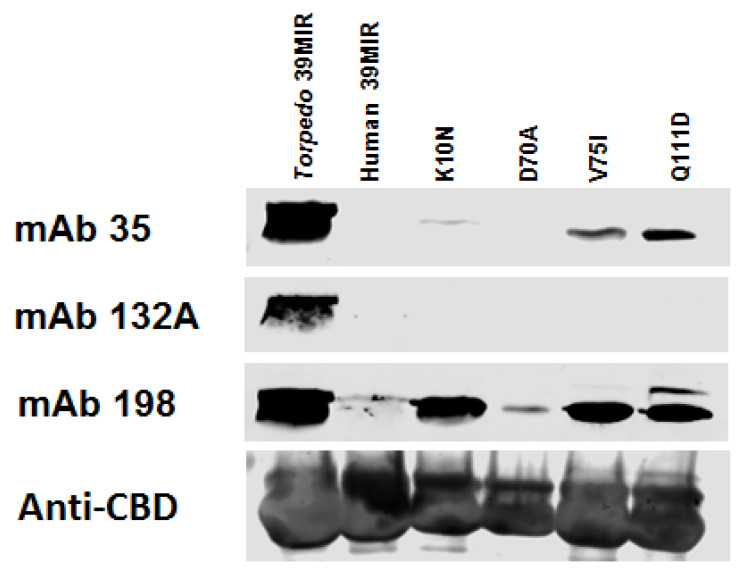
Western blots of the four single point mutated human 39MIR mimics back to the *Torpedo* MIR aa. Chitin beads adsorbed with the *Torpedo* 39MIR, human 39MIR and four mutant human 39MIR peptide-IChBD fusion proteins were all run on an SDS-PAGE gel and transferred to a nitrocellulose membrane and analyzed for binding mAbs 35, 132A, and 198, as well as an anti-ChBD mAb, using IRDye 680 goat anti-rat IgG. The first two lanes here are the two lanes of Figure 3.

The H39MIR-IChBD mimic was then site-specifically mutated, singly incorporating each of the four *Torpedo* α subunits’ aas (Hto*T*), K10N, D70A, V75I, and Q111D, to identify the contribution of each of these aas to the building of MIR mimic binding of mAbs 35 and 132A and the enhancing of the affinity of mAb 198 (Figure 6). Two of the single site-mutated peptide constructs, V75I and Q111D, of the H39MIR restored binding by mAb 35, albeit to a very low level compared to it’s binding to the *T*39MIR construct. In addition, the D70A mutant also failed to bind mAb 35 or 132A, and left mAb 198 binding about as phlegmatically impaired as the binding to the unmutated H39MIR. In contrast, each of the K10N, V75I, and Q111D mutations greatly enhanced mAb 198 binding compared to its binding to the unmutated H39MIR. Taken individually, these four single point mutations do not build mAb binding to the chimeric *T*/H39MIRs to the level seen for the *T*39MIR. The best mAb binding heading to that of the *T*39MIR construct for the T/H39MIR chimeric mimics likely results from a combination of two or more of the reverse mutations that were poison to mAb binding to the H/*T*39MIR chimeric mutants (Figure 5b).

**Figure 7:**
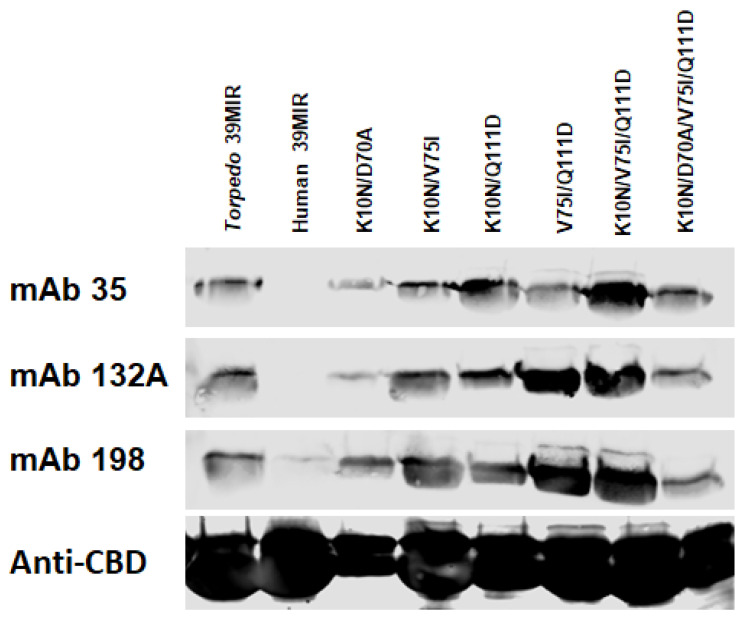
Western blots of multiply mutated human 39MIRs. Chitin beads adsorbed with the *Torpedo* 39MIR, human 39MIR, and various mutant human 39MIR peptide-IChBD fusion proteins were all run on an SDS-PAGE gel and transferred to a nitrocellulose membrane and analyzed for binding mAbs 35, 132A, and 198. as well as an anti-ChBD mAb, using IRDye 680 goat anti-rat IgG. These fusion proteins were expressed with pTXB1.

To explore those possibilities, various double *Torpedo* mutants of the H39MIR construct were produced and assayed for mAb binding. The K10N/V75I, K10N/Q111D, and V75I/Q111D double mutants exhibit binding of mAbs 35, 132A, and 198 to about the level of, or better than, the level of their binding to the *T*39MIR construct (Figure 7 and Table 2). The K10N/D70A double mutant has the least effect on enhancing mAb binding, at least compared to that of *T*39MIR (31% for mAb 35, 16% for mAb 132A, and 72% for mAb 198). The K10N/V75I/Q111D triple mutant (3XH39MIR) combines the best double mutant, V75I/Q111D, with the K10N mutation, and that construct binds 2.5–3 times more mAbs 35, 132A, and 198 than the *T*39MIR does. Finally, the quadruple mutant peptide (4XH39MIR) containing all four mutations (K10N, D70A, V75I, and Q111D) binds mAbs 132A and 198 at levels comparable to those by *T*39MIR, and is recognized by 1.7 times more mAb 35 than *T*39MIR (Table 2).

### 2.5. Abs in MG Sera Bind to the Triple and Quadruple Chimeric T/H39MIR Peptides

**Figure 8:**
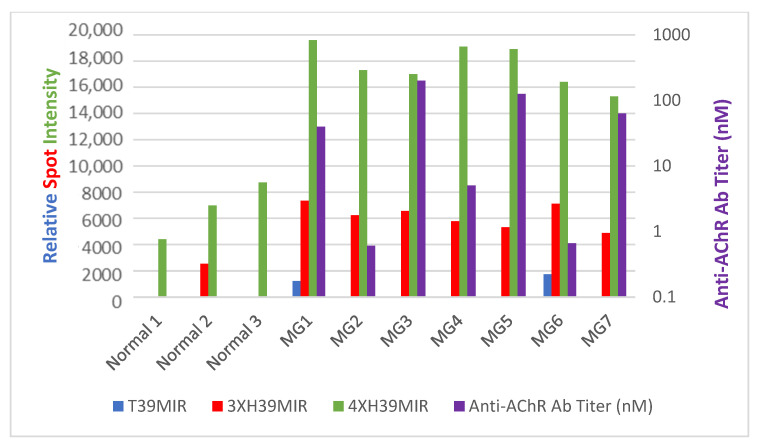
Abs in MG sera bound to 3XH39MIR and 4XH39MIR mutants in dot blots. A total of 2.5 μg of His-tagged 3XH39MIR (red), 4XH39MIR (green), T39MIR (blue), and H39MIR (data not presented) were spotted and probed with normal and MG human sera at 1/100,000 dilution. Bound Abs were detected with IRDye 680 goat anti-human IgG. The serum dilution was determined by the dilution of normal human serum that shows no binding to 2.5 μg of 3XH39MIR-hexahis peptide. Hence, 1/100,000 is the dilution that is used for the MG sera. The anti-AChR Ab titer in nM in the human sera is also plotted (purple) on a log scale, as indicated on the right vertical axis.

Given that the two chimeric peptides, 3XH39MIR and 4XH39MIR, enhance anti-MIR mAb binding to levels comparable to or better than those of the *T*39MIR construct, we next assayed their binding to anti-MIR Abs in MG serum. We prepared HexaHis-tagged peptides of *T*39MIR, H39MIR, and both 3XH39MIR and 4XH39MIR chimeric peptides, expressed them, purified them, and probed them for binding of anti-MIR Abs from human MG sera at a 1/100,000 dilution in dot blots of 2.5 μg of the purified His-tagged peptides. The results are presented in Figure 8, along with the anti-AChR titers of the human sera. An anti-AChR titer of >0.4 nM is positive for MG. The *T*39MIR is recognized by Abs in only two of the seven MG sera at very low levels, and the H39MIR is not recognized by Abs from any MG serum Abs. However, both 3XH39MIR and 4XH39MIR His-tagged peptides are recognized by Abs in all tested MG sera. 4XH39MIR was recognized by Abs in three normal human sera as well, whereas the 3XH39MIR was recognized by a low level of Abs in only one of the normal sera (Figure 8). These two chimeric MIR mimic peptides bind to the combining sites of many if not all MIR-directed Abs in MG sera that are the major pathogenic component in MG patients’ sera. As such, the peptides have diagnostic and therapeutic potential in treating MG.

### 2.6. Clearing Anti-MIR Abs from EAMG Serum with 3XH39MIR-IChBD/Chitin Beads

**Figure 9:**
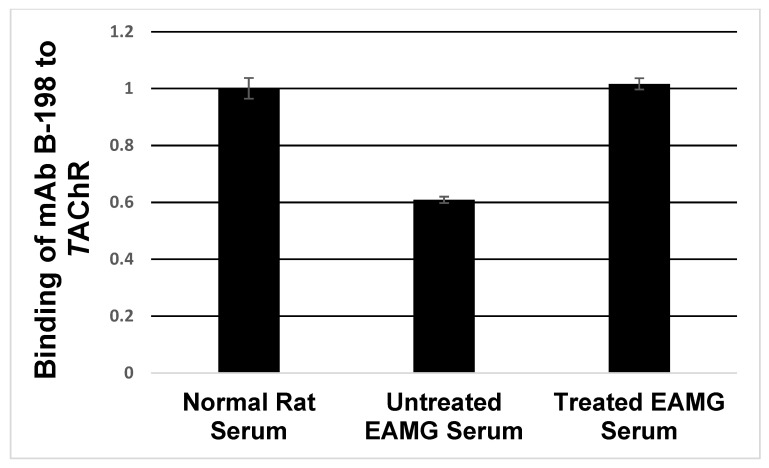
Removal of pathogenic anti-MIR Abs in EAMG serum. Anti-MIR Abs block the binding of mAb B-198 to *T*AChRs (untreated EAMG) detected with the A_450_ from HRP-streptavidin binding to the biotin and developed with phenylenediamine/H_2_O_2_. EAMG serum is cleared of anti-MIR Abs using column-bound 3XH39MIR-IChBD/Chitin beads. Treated EAMG serum now lacks anti-MIR Abs to block mAb B-198 binding thus restoring mAb B-198 binding to control (PBS and normal rat serum) levels. N = 3, ±SEM.

Since His-tagged 3XH39MIR is recognized by Abs in MG sera, the 3XH39MIR-IChBD-chitin bead preparation was column tested for removing anti-MIR Abs from EAMG serum, as was performed with *T*39MIR-IChBD in [17]. In the current experiment, 3X39MIR-IChBD is used to clear anti-MIR Abs from EAMG serum, and mAb biotin labeled 198 (B-198) binding was used to quantitate the accessible MIR sites on the *T*AChR with the biotin detected by HRP-streptavidin developed with phenylenediamine/H_2_O_2_ followed by an A_450_ reading. Columns 1 and 2 of Figure 9 illustrate that 40% of the MIR sites are blocked by anti-MIR Abs from an EAMG serum. Passing that EAMG serum through the 3XH39MIR-IChBD-chitin bead column, and then testing its MIR blocking ability again (column 3), shows that very few, if any, of the MIR sites blocked previously are blocked by the treated EAMG serum. The anti-MIR Abs have been removed by the 3XH39MIR-IChBD construct.

## 3. Discussion

*A human anti-AChR MIR Ab binding peptide.* With the goal to produce a diagnostic/therapeutic peptide mimic of the human AChR MIR, we started with producing the H39MIR mimic. However, it does not bind the anti-MIR mAbs as well as the *T*39MIR mimic, even though the mAbs bind to the human and other species’ intact neuromuscular AChR MIRs. Furthermore, it does not seem to bind MIR-directed Abs from MG sera. The eight aa differences of the human MIR mimic sequence compared to that of the *T*39MIR sequence result in the H39MIR mimic not binding the anti-MIR mAbs as well as the *T*39MIR mimic does.

Comparing the structures of the *Torpedo* MIR to the mouse MIR possessing 7 of the 8 human mutations (Figure 4) reveals some striking differences that could account for the mAbs’ reluctance to bind to the H39MIR mimic. The three MIR segments in the *Torpedo* α-subunit structure are more loosely packed than those in the mouse structure, and the *Torpedo* N-terminal helix seems more loosely associated with the cluster than the mouse N-terminal helix does (Figure 4). Consistent with this view is the structural insight from the surface area of contact between the three segments in the two models: for the *Torpedo* MIR the total intra surface area of contact is 474.7 Å^2^, whereas for the mouse, it is 832.9 Å^2^ [19].

Additionally, there are striking differences in orientation and accessibility of critical MIR aas. For example, in the first MIR segment H3 and R6 sit like crown jewels on top of the *Torpedo* MIR, whereas in the mouse MIR, they are more compactly folded onto the central segment (Figure 4). The central MIR segment mapped to the mouse AChR α(61–76) [20], and subsequently refined to the *Torpedo* and human α(67–76) [5], is both tightly packed and surface-accessible in both the *Torpedo* and mouse structures. However, their structural arrangement in the folded structures is quite different. For example, the hydroxyl group of Y72 at the center of the core segment is essential for MIR-directed mAb 132A recognition [15]. The binding of mAb 132A is dramatically decreased to the *Torpedo* mutant Y72F [15]. In the *Torpedo* segment, Y72 prominently protrudes from the external side of the MIR complex (Figure 4) whereas in the mouse core it is completely buried, which alone could explain the lack of binding of mAb 132A to the H39MIR mimic. It is likely that one or more of the different aas in the mammalian MIR mimics give rise to a slightly more tightly folded structure so that the MIR mimic peptide more closely resembles that in the mammalian α-subunit extracellular domain crystal structure [19] than that in the *Torpedo* relaxed MIR structure that presents binding sites for mAbs 35 and 132A, as well as 198.

Assessing which of the eight H39MIR mimic mutations are most deleterious for anti-MIR mAb binding to H/*T*39MIR chimeric mimics reveals four mutations that have negligible effects on mAb binding and four mutations that reduce mAb binding: N10K, A70D, I75V, and D111Q. These four were judged to be the most inhibitory mutations (Figure 5b). To induce binding of the anti-MIR mAbs to H39MIR, the H39MIR amino acids that inhibit the binding of mAbs to chimeric H/*T*39MIR mimics were replaced individually with the respective four *Torpedo* aas (Figure 6). Since none of the individual *Torpedo* aas provided mAb binding by the T/H39MIR chimeric mutants to the level of the *T*39MIR mimic, pairs of mutants were tested, and Figure 7 shows the best binding of pairs along with the triple and quadruple H39MIR chimeric mutants, 3XH39MIR and 4XH39MIR, respectively. Three of the paired mutants can bind the mAbs as well as *T*39MIR, but those that include V75I and Q111D show the best binding. But the best of all is the K10N/V75I/Q111D triple mutant (3XH39MIR) that binds 2.5–3 times the amount of the mAbs as that of the *T*39MIR mimic construct (Table 2). The K10N/D70A/V75I/Q111D quadruple mutant (4XH39MIR) binds roughly the equivalent amount of mAb as that of the *T*39MIR for the three mAbs. Based on mAb binding, the 3XH39MIR is the choice for a human MIR mimic. It is likely that the 2.5–3 times higher mAb binding of 3XH39MIR is reflective of the fraction of the mimics correctly refolding after SDS denaturation. So, the goal is to build a therapeutic with the targeting agent consisting of one of our mutant peptide MIR mimics.

*Potential as a diagnostic assay.* Given the excellent mAb binding to the chimeric *T/*H39MIR triple and quadruple mutant mimic peptides (3XH39MIR and 4XH39MIR), we next assayed these for binding of anti-MIR Abs from MG serum. Seven MG sera were tested at a 1/100,000 dilution for binding to dot blots of 2.5 μg of the His-tagged peptides that have not been SDS denatured. Figure 8 displays the titer (purple) of the anti-AChR Ab in patient sera on a log scale as well as their binding to the non-denatured 3X (red), 4X (green), *T*39MIR (blue) mimics on a linear scale. (H39MIR data is negative and not displayed). Although better recognized by anti-MIR directed mAbs, the 3XH39MIR mimic binds less MG serum Abs than the 4XH39MIR mimic. However, it only binds low amounts of Abs in one of three normal human sera, unlike the 4XH39MIR mimic that binds more Abs in two of the normal sera than in the MG sera to the 3XH39MIR peptide. In a curious result, two of the human MG sera have components that bind to the *T*39MIR mimic (blue), but none of the sera have components that bind to the H39MIR mimic. The binding observed in Figure 8 to the chimeric mutant mimics demonstrates their potential for being the basis of a sensitive clinical anti-MIR Ab assay that could assess potential correlation of anti-MIR Ab titer with disease severity as well as with the effectiveness of various therapeutic pathways [21]. It is also evident that sera anti-MIR binding components are more constant for these sick MG patients than their anti-AChR Ab titers. (Recall in Figure 8 the anti-AChR Ab titers are on a log scale and the anti-MIR binding Ab quantitation is on a linear scale.)

*Potential as an MG therapeutic*. The clearing of EAMG serum of anti-MIR Abs by 3XH39MIR-IChBD-chitin beads (Figure 9) in vitro demonstrates the potential of these constructs to clear anti-MIR Abs from EAMG and MG serum. By attaching the 3XH39MIR peptide to the N-terminus of the hinge-C_H_2-C_H_3 of the human IgG1 heavy chains may provide a therapeutic anti-idiotype-like pseudo-Ab construct to treat MG. The three potential properties of this type of F_c_-construct, anti-MIR Ab blockade, increased anti-MIR Ab clearance, and perhaps selective anti-MIR memory B cell elimination, give hope that these constructs potentially represent a non-steroidal prototypic biologic agent for antigen-specific editing of the immune systems of MG patients. In addition, using the chimeric mutant peptide mimics in construction of a T cell CAAR to target pathogenic B cells for elimination may provide additional therapeutic avenues [22,23,24,25].

One potential downside of a biologic therapeutic built from our MIR mimics is the immune system may find with the MIR mimic a further antigenic stimulation. However, since the peptides are not congregated together in mass such as on a virus or bacterium, it is likely that at the low concentration required to soak up anti-AChR MIR Abs at 7–100 nM, our doses may be low enough to avoid further antigenic stimulation of the Abs that they are targeting for removal. Furthermore, they will also bind to and eliminate any new MIR directed Abs they create. The other potential shortcoming of our approach is the long-lived plasma cells (LLPCs) that lack surface antigen receptors while still producing anti-AChR MIR Abs. However, the therapeutic will remove the secreted pathogenic Abs. The timing of quantification of dosing to keep those Abs in check is yet to be worked out, but our therapeutic biologic is designed to block and remove those Abs as well. Currently used therapeutics may affect the production of Abs from the LLPCs, but it is not clear how well-understood Ab production from this source is affected by the alternate therapeutics.

The advantage of our therapeutic biologic compared to virtually all current therapeutics is the specificity compared to existing therapies. For example, rituximab directed to the CD20 of B cells exhibits no specificity for the anti-MIR specific B cells and will target all CD20 B cells, thus altering the functioning of the immune system globally. Most of the other MG therapies suffer from the same lack of specificity. There is much to recommend a non-steroidal, antigen-specific biologic approach, and we have created the targeting element of such an approach. We are proceeding with the in vivo testing of the 3XH39MIR-rat IgG1 construct for in vivo lifetime, side effects, and therapeutic effectiveness of the biologic therapeutic in EAMG.

## 4. Materials and Methods

### 4.1. Plasmid Construction

The plasmid construction of pTYB1-*Torpedo* 39MIR (for *T*39MIR) and the expression and purification of peptides are described in [17]. The construction of pTXB1-human 39MIR (for H39MIR) and pTXB1-mouse 39MIR (for rat/mouse 39MIR) was performed in the same manner, using the pTXB1 plasmid (New England Biolabs, Rowley, MA, USA). A list of the oligomers used can be found in the Supplementary Material.

### 4.2. Mutagenesis

Primers (f:forward, r:reverse) were used to introduce the point mutations into the *pTYB1-Torpedo 39MIR* and *pTXB1-human 39MIR sequences* by site-directed mutagenesis with the QuikChange Site-Directed Mutagenesis Kit (Stratagene, Cedar Creek, TX, USA). For pairwise and more point mutations, the appropriate combination of primers was used. The presence of the correct mutation was verified by sequencing. A list of oligomers used can be found in Supplementary Material (Integrated DNA Technologies, San Diego, CA, USA).

### 4.3. Western Blots

An equal volume of 2× non-reducing sample buffer was added to the chitin beads (New England Biolabs, Rowley, MA, USA), with the various MIR peptides adsorbed and then heated at 100 °C for 5 min. Equimolar peptides were loaded onto an 8% SDS-PAGE gel. Protein was transferred to a Trans-Blot Transfer Medium nitrocellulose membrane (Bio-Rad, Hercules, CA, USA). Transfer was performed at 4 °C for 1 h at a constant 100 V. Blots were blocked with 5% non-fat dry milk in PBS for 1 h at 25 °C. Blots were then probed with our anti-*T*AChR mAb library and diluted 1:2 in 5% non-fat dry milk in PBS for 1 h at 25 °C, followed by incubation with IRDye 680 goat anti-rat IgG (Licor, Lincoln, NE, USA), which was diluted 1:5000 in PBS for 1 h at 25 °C. The membrane was washed three times for 5 min in PBS and imaged using the Odyssey CLx Imaging System.

### 4.4. MG Patient Blood Collected in Accordance with the Ethical Approval of the UC Davis Review Board

Blood from patients with and without MG was collected. After clotting, serum was prepared by centrifugation at 1000–2000× *g* for 10 min in a refrigerated centrifuge. The resulting serum was immediately frozen at −20 °C or lower and stored for later use.

### 4.5. Dot Blots

Multiple strips of Trans-Blot Transfer Medium nitrocellulose membrane (Bio-Rad, Hercules, CA, USA) were each spotted with 2.5 µg of the three his-tagged MIR mimic peptides, 3XH39MIR, 4XH39MIR, and T39MIR. The membranes were dried and blocked with 5% milk in PBS for 1 h at 25 °C followed by incubation with normal human (control) or MG patient sera diluted to 1:100,000 in 5% non-fat dry milk in PBS for 1 h at 25 °C. The serum dilution was determined by the dilution of normal human sera that no longer show any Ab binding to 2.5 μg of 3XH39MIR-hexahis peptide. Hence, the negative control is that of the 1/100,000 diluted normal human sera showing no anti-MIR Ab binding to 3XH39MIR, and this is the dilution that was used for the MG sera. The membrane was washed for 5 min three times with PBS and incubated with IRDye 680 goat anti-human IgG (Licor, Lincoln, NE, USA) for 1 h at 25 °C. The membrane was washed three times for 5 min in PBS and imaged using the Odyssey CLx Imaging System (Licor, Lincoln, NE, USA).

### 4.6. His−Tagged Peptide Expression and Purification

*E. coli* BL(DE3) (Novagen, Vadodara, India) containing pET28b-*Torpedo*, pET28b-human, pET28b-TripleMut, and pET28b-QuadMut was grown at 37 °C until the OD_600_ reached 0.5–0.8, after which 0.5 mM isopropyl β-D thiogalactoside (IPTG) was added and the culture was grown at 25 °C for 12–14 h. The cell pellet of a 1 L culture was resuspended in 25 mL of the column buffer (20 mM Tris-HCL, pH8, 500 mM NaCl, 1 mM EDTA). After sonication on ice for 10 min, the cell debris was removed by centrifugation at 20,000× *g* for 30 min. His-tagged peptides were purified using Profinity IMAC Resins (Bio-Rad, Hercules, CA, USA).

### 4.7. Western Blot Analysis

Fluorescent band intensities from the mAb probed blots detected with IRDye680 goat anti-rat IgG were measured using Image Studio software (Li-Cor, version 6.0). The mAb band intensities were divided by the band intensity of the anti-chitin-binding domain loading control to obtain the ratio of the mAb/anti-chitin binding domain. The mAb35-*T*39MIR band/anti-chitin binding domain band is considered 1, and all other ratios are compared to it to determine the relative increase or decrease in binding due to mutations.

### 4.8. Surface Area Analysis

The surface area of contact between the three segments in the *Torpedo* MIR and in the mouse MIR is determined as described in [18].

### 4.9. Clearing of EAMG Serum

ELISA microtiter plates were coated with 5 × 10^−9^ M of alkali-stripped membrane-bound *T*AChR in gradient buffer (8 mM Na_2_HPO_4_, 2 mM NaH_2_PO_4_, 1 mM EDTA, 1 mM EGTA, 0.025% NaN_3_) overnight at 4° C, followed by blocking with 1% BSA in PBS for 1 h at 25 °C. Three wells were treated with 100 µL of PBS, three wells with normal rat serum were diluted 1:100 with PBS, and three wells were treated with EAMG serum, diluted 1:100 with PBS. Another three wells were treated with EAMG serum diluted 1:100 with PBS and run four times over spin columns containing 1.5 mL of the 39MIR-IChBD/chitin bead construct, incubating the beads with the effluent in each cycle for 1 h. Finally, 100 µL of the last effluent was added to each of three wells of the coated ELISA plate, and all wells were incubated for 1 h. The treated wells were washed six times with PBS, and finally, 100 μL of 20 mg/mL biotinylated mAb 198 (B-198) was added to the wells and incubated for 1 h at 25 °C. Plates were then washed three times with PBS and the wells were incubated for 1 h with HRP-conjugated streptavidin (ICN Biomedicals, Inc., Costa Mesa, CA, USA), followed by 5 washes with PBS. 100 µL of 1 mg/mL o-phenylenediamine dihydrochloride (Sigma, St. Louis, MO, USA) in 0.1 M citrate buffer (pH 4.5) with 0.03% H_2_O_2_ was added to each well and the absorbance was read at 450 nm on a FilterMax F5 Microplate Reader (Molecular Devices, San Jose, CA, USA). When the absorbance of the control wells (wells treated with PBS) reached a value between 1.0 and 1.5, the entire plate was read. The average absorbance from the wells treated with PBS was used as the control to measure the amount of MIR detected by the A_450_ of the B-198/HRP-streptavidin system. The average absorbance values from the wells of each set of additives were divided by average of those from the control wells, and the average values and standard deviations from all the additives were calculated.

## Figures and Tables

**Table 2:** Quantitative summary of Western blots of multiple site-directed mutated H39MIR-constructs. (- no binding). Each entry for binding to a mutated construct is the ratio of mAb stain density from the mutation to the stain density of the mAb binding to the *T*39MIR-construct.

Mutant	mAb 35	mAb 132A	mAb 198
*T*39MIR	1.00	1.00	1.00
H39MIR	-	-	0.09
K10N/D70A	0.31	0.16	0.72
K10N/V75I	1.10	1.25	1.79
K10N/Q111D	2.31	1.07	1.19
V75I/Q111D	0.92	3.34	2.71
K10N/V75I/Q111D	2.97	2.46	2.52
K10N/D70A/V75I/Q111D	1.70	0.99	0.99

## Data Availability

Data is contained within the article and Supplementary Materials.

## Supplementary Materials

The following supporting information can be downloaded at: https://www.mdpi.com/article/10.3390/ijms26010229/s1.
