# Supplementary material for: Development of Peptide Mimics of the Human Acetylcholine Receptor Main Immunogenic Region for Treating Myasthenia Gravis
Source: Int J Mol Sci. 2024 Dec 30;26(1):229. doi: 10.3390/ijms26010229 (PMC11719904; doi:10.3390/ijms26010229)
Supplement: Supplementary file 1 [file ijms-26-00229-s001.zip › ijms-3364037-supplementary.pdf]

2.1. Plasmid construction
pTXB1-Human39MIR1 TATGTCTGAACATGAAACACGTTTGGTTGCTAAATTATTTGG
pTYB1-Human39MIR2 GCCACCAAATAATTTAGCAACCAAACGTGTTTCATGTTCAGACA
pTXB1-Human39MIR3 TGGCGGCTCTCTTAAATGGAATCCAGATGATTATGGTGGA
pTXB1-Human39MIR4 TAACTCCACCATAATCATCTGGATTCCATTTAAGAGAGCC
pTXB1-Human39MIR5 GTTAAAAAGATCCATGGCTCTTTGCAGTATACGGGACAT
pTXB1-Human39MIR6 GCAATGTCCCGTATACTGCAAAGAGCCATGGATCTTTT
pTXB1-Mouse39MIR1 TATGTCTGAACATGAAACACGTTTGGTTGCTAAATTATTTGG
pTXB1-Mouse39MIR2 ACAGACTTGTACTTTGTGCAAACCAACGATTTAATAAACCACCG
pTXB1-Mouse39MIR3 TGGCGGCTCTCTTAAATGGAATCCAGATGATTATGGTGGA
pTXB1-Mouse39MIR4 TAACTCCACCATAATCATCTGGATTCCATTTAAGAGAGCC
pTXB1-Mouse39MIR5 GTTAAAAAGATCCATGGCTCTTTGGATTATACGGGACAT
pTXB1-Mouse39MIR6 GCAATGTCCCGTATAATCCAAAGAGCCATGGATCTTTT

For various peptides with a C-terminal His-tag, the following oligomers were cloned into the plasmid pET-28b (Novagen) similar to pTXB1. The construct coded for a His-tag that added 8 aa residues (VLHHHHHH) to the C-terminus of these peptides:

pET28b-Torpedo39MIR1 CATGTCTGAACATGAAACACGTTTGGTTGCTAATTTATTAGG
pET28b-Torpedo39MIR2 GCCACCTAATAAATTAGCAACCAAACGTGTTTCATGTTCAGA
pET28b-Torpedo39MIR3 TGGCGGCTCTCTTCGCTGGAATCCAGCCGATTATGGTGGA
pET28b-Torpedo39MIR4 TAATTCCACCATAATCGGCTGGATTCCAGCGAAGAGAGCC
pET28b-Torpedo39MIR5 ATTAAAAAGATCAGAGGCTCTTTGGATTATACGGGAAAAC
pET28b-Torpedo39MIR6 TCGAGGCATTTTCCCGTATAATCCAAAGAGCCTCTGATCTTTT
pET28b-Human39MIR1 CATGTCTGAACATGAAACACGTTTGGTTGCTAAATTATTTGG
pET28b-Human39MIR2 GCCACCAAATAATTTAGCAACCAAACGTGTTTCATGTTCAGACA
pET28b-Human39MIR3 TGGCGGCTCTCTTAAATGGAATCCAGATGATTATGGTGGA
pET28b-Human39MIR4 TAACTCCACCATAATCATCTGGATTCCATTTAAGAGAGCC
pET28b-Human39MIR5 GTTAAAAAGATCCATGGCTCTTTGCAGTATACGGGACATC
pET28b-Human39MIR6 TCGAGATGTCCCGTATACTGCAAAGAGCCATGGATCTTTT
pET28b-TripleMut39MIR1 CATGTCTGAACATGAAACACGTTTGGTTGCTAACTTATTTGG
pET28b-TripleMut39MIR2 GCCACCAAATAAGTTAGCAACCAAACGTGTTTCATGTTCAGACG
pET28b-TripleMut39MIR3 TGGCGGCTCTCTTAAATGGAATCCAGATGATTATGGTGGA
pET28b-TripleMut39MIR4 TAATTCCACCATAATCATCTGGATTCCATTTAAGAGAGCC
pET28b-TripleMut39MIR5 ATTAAAAAGATCCATGGCTCTTTGGATTATACGGGACATC
pET28b-TripleMut39MIR6 TCGAGATGTCCCGTATAATGCAAAGAGCCATGGATCTTTT
pET28b-QuadMut39MIR1 CATGTCTGAACATGAAACACGTTTGGTTGCTAACTTATTTGG
pET28b-QuadMut39MIR1 GCCACCAAATAAGTTAGCAACCAAACGTGTTTCATGTTCAGACG
pET28b-QuadMut39MIR1 TGGCGGCTCTCTTAAATGGAATCCAGCGGATTATGGTGGA
pET28b-QuadMut39MIR1 TAATTCCACCATAATCCGCTGGATTCCATTTAAGAGAGCC
pET28b-QuadMut39MIR1 ATTAAAAAGATCCATGGCTCTTTGGATTATACGGGACATC
pET28b-QuadMut39MIR1 TCGAGATGTCCCGTATAATGCAAAGAGCCATGGATCTTTT

2.2. Mutagenesis

2.2.1. pTYB1-Torpedo 39MIR

N10Kf CGTTTGGTTGCTAAATTATTAGGTGGCGGC
N10Kr GCCGCCACCTAATAATTTAGCAACCAAACG
L12F1f GGTTGCTAATTTATTTGGTGGCGGCTCTCTTC
L12F1r GAAGAGAGCCGCCACCAAATAAATTAGCAACC
A70Df GCTCTCTTCGCTGGAATCCAGATGATTATGGTGGAATTAAAAAG
A70Dr CTTTTTAATTCCACCATAATCATCTGGATTCCAGCGAAGAGAGC
I75Vf GCCGATTATGGTGGAGTTAAAAAGATCAGAGG
I75Vr CCTCTGATCTTTTTAACTCCACCATAATCGGC
R66Kf GGTGGCGGCTCTCTTAAATGGAATCCAGCCGATTATGG
R66Kr CCATAATCGGCTGGATTCCATTTAAGAGAGCCGCCACC
R79Hf GGAATTAAAAAGATCCATGGCTCTTTGGATTATACGG
R79Hr CCGTATAATCCAAAGAGCCATGGATCTTTTTAATTCC
D111Qf GATCAGAGGCTCTTTGCAGTATACGGGAAAATGC
D111Qr GCATTTTCCCGTATACTGCAAAGAGCCTCTGATC
K115Hf CTCTTTGGATTATACGGGCATATGCTTTGCCAAGGGTAC
K115Hr GTACCCTTGGCAAAGCATATGCCCGTATAATCCAAAGAG

2.2.2. pTXB1-human 39MIR

K10Nf CGTTTGGTTGCTAATTTATTTGGTGGCGGC
K10Nr GCCGCCACCAAATAAATTAGCAACCAAACG
D70Af TGGAATCCAGCAGATTATGGTGGA
D70Ar TCCACCATAATCTGCTGGATTCCA
V75If TATGGTGGAATTAAAAAGATCCATGGCTCT
V75Ir AGAGCCATGGATCTTTTTAATTCCACCATA
Q111Df CATGGCTCTTTGGATTATACGGGACAT
Q111Dr ATGTCCCGTATAATCCAAAGAGCCATG
